# Broadening the Genetic Spectrum of Painful Small-Fiber Neuropathy through Whole-Exome Study in Early-Onset Cases

**DOI:** 10.3390/ijms25137248

**Published:** 2024-06-30

**Authors:** Kaalindi Misra, Milena Ślęczkowska, Silvia Santoro, Monique M. Gerrits, Elisabetta Mascia, Margherita Marchi, Erika Salvi, Hubert J. M. Smeets, Janneke G. J. Hoeijmakers, Filippo Giovanni Martinelli Boneschi, Massimo Filippi, Giuseppe Lauria Pinter, Catharina G. Faber, Federica Esposito

**Affiliations:** 1 Laboratory of Human Genetics of Neurological Disorders, IRCCS San Raffaele Scientific Institute, Institute of Experimental Neurology, 20132 Milan, Italy; 2 Department of Toxicogenomics, Maastricht University, 6229 ER Maastricht, The Netherlands; 3 Department of Neurology, Mental Health and Neuroscience Research Intsitute, Maastricht University Medical Centre+, 6229 ER Maastricht, The Netherlands; 4 Department of Clinical Genetics, Maastricht University Medical Centre+, 6229 HX Maastricht, The Netherlands; 5 Neuroalgology Unit, Fondazione IRCCS Istituto Neurologico Carlo Besta, 20133 Milan, Italy; 6 Aldo Ravelli Center for Neurotechnology and Experimental Brain Therapeutics, Department of Health Sciences, University of Milan, 20142 Milan, Italy; 7 Clinical Neurology Unit, Azienda Socio-Sanitaria Territoriale Santi Paolo e Carlo and Department of Health Sciences, University of Milan, 20142 Milan, Italy; 8 Neurology and Neurorehabilitation Unit, IRCCS San Raffaele Scientific Institute, 20132 Milan, Italy; 9 Vita-Salute San Raffaele University, 20132 Milan, Italy; 10 Neurophysiology Service, IRCCS San Raffaele Scientific Institute, 20132 Milan, Italy; 11 Neuroimaging Research Unit, Division of Neuroscience, IRCCS San Raffaele Scientific Institute, 20132 Milan, Italy; 12 Department of Biomedical and Clinical Sciences “Luigi Sacco”, University of Milan, 20157 Milan, Italy

**Keywords:** Small-Fiber Neuropathy, Early-Onset, whole-exome study, neuropathic pain, genetics

## Abstract

Small-Fiber Neuropathy (SFN) is a disorder of the peripheral nervous system, characterised by neuropathic pain; approximately 11% of cases are linked to variants in Voltage-Gated Sodium Channels (VGSCs). This study aims to broaden the genetic knowledge on painful SFN by applying Whole-Exome Sequencing (WES) in Early-Onset (EO) cases. A total of 88 patients from Italy (n = 52) and the Netherlands (n = 36), with a disease onset at age ≤ 45 years old and a Pain Numerical Rating Score ≥ 4, were recruited. After variant filtering and classification, WES analysis identified 142 potentially causative variants in 93 genes; 8 are Pathogenic, 15 are Likely Pathogenic, and 119 are Variants of Uncertain Significance. Notably, an enrichment of variants in transient receptor potential genes was observed, suggesting their role in pain modulation alongside VGSCs. A pathway analysis performed by comparing EO cases with 40 Italian healthy controls found enriched mutated genes in the “Nicotinic acetylcholine receptor signaling pathway”. Targeting this pathway with non-opioid drugs could offer novel therapeutic avenues for painful SFN. Additionally, with this study we demonstrated that employing a gene panel of reported mutated genes could serve as an initial screening tool for SFN in genetic studies, enhancing clinical diagnostics.

## 1. Introduction

Small-Fiber Neuropathy (SFN) arises from damage to small nerve fibers, specifically the Aδ and demyelinated C fibers, which transmit sensory signals and regulate autonomic functions. SFN is a common disorder with a prevalence of 131.5 per 100,000 inhabitants [1]. It presents with diverse symptoms, including sensory issues and autonomic dysfunction, with neuropathic pain (NP) being the prominent feature [2,3]. Diagnosing SFN poses a challenge due to the variable intensity and distribution of symptoms, which predominantly affect the lower extremities and may extend to the upper limbs. Routine clinical examinations often fail to detect abnormalities, leading to potential underdiagnosis of SFN [3,4]. Therefore, advanced diagnostic techniques such as Intra-Epidermal Nerve Fiber Density (IENFD) determination through skin biopsy and temperature threshold testing (TTT) are used for a precise diagnosis [5,6].

Advancements in sequencing technologies have identified heterozygous mutations in Voltage-Gated Sodium Channel (VGSC) genes, such as SCN9A-SCN11A, which contribute to pain amplification in SFN patients through an autosomal dominant inheritance pattern. However, these mutations account for only 11% of SFN cases; this percentage could rise to 18.1% with the inclusion of additional VGSC genes like SCN3A, SCN7A-SCN8A, and SCN1B-SCN4B in screening processes [7,8]. Recent reviews and research suggest that other genetic factors, including transient receptor potential (TRP) cation channels, potassium voltage-gated channels, hyperpolarization-activated and cyclic nucleotide-gated channels, Ca^2+^-activated Cl^−^ channels, and Collagen type VI alpha-5, also play a role in pain or itch modulation in SFN [8,9,10,11,12]. These findings indicate the complex genetic landscape of SFN and the need for comprehensive genetic analyses.

To broaden the genetic determinants of SFN, we decided to focus on Early-Onset (EO) cases, who are of particular interest because they suggest the existence of a significant predisposing genetic burden, inducing SFN clinical signs and symptoms at a young age [13]. Whole-Exome Sequencing (WES) was employed to examine protein-coding exonic regions, potentially revealing novel genetic variants influencing SFN susceptibility. The primary aim of the study is to enhance knowledge of genetic mutations associated with SFN, advancing our understanding of its complex genetic pathophysiology.

## 2. Results

### 2.1. Patient Characteristics/Cohort Phenotype

**Figure 1:**
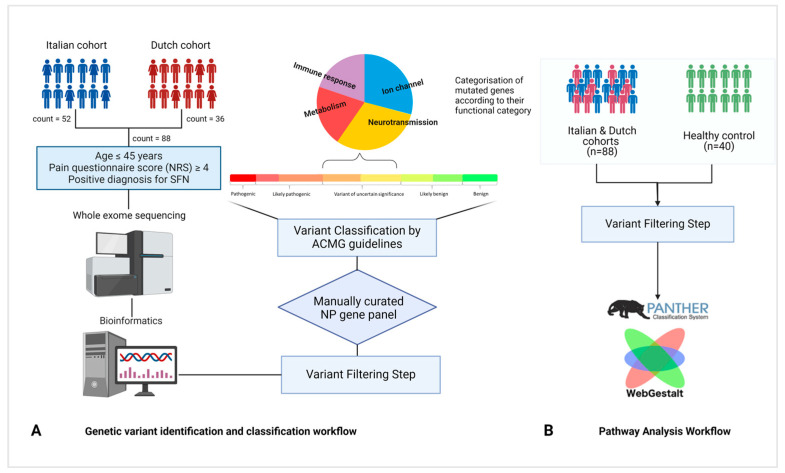
A schematic representation of patient cohorts and study workflow. (**A**) Pipeline applied for variant identification and classification. (**B**) Pathway analysis workflow.

Eighty-eight individuals from Italy and the Netherlands, with EO-SFN, were included in the study as shown in Figure 1. All of them had features of NP, with a Pain Numerical Rating Score (NRS) ≥ 4. The study population included 63.6% of females and the average age at onset was 33.3 years old.

All the Italian patients had reduced IENFD; in the Netherlands cohort, twenty-two (n = 22, 61%) patients had reduced IENFD, and fourteen (n = 14, 39%) had normal IENFD. Most of the patients from the Netherlands (n = 24, 75%) had abnormal TTT, while only eight (25%) patients had normal TTT values; in four patients TTT was not performed. Demographic and clinical features of the studied cohorts are reported in Table 1.

### 2.2. Identification of Genetic Variants

In this study, we performed WES experiments to identify genetic variants in EO-SFN patients. The quality of the obtained data was evaluated based on several aspects as mean coverage per sample, coverage of the target regions per sample, and the transition–transversion (Ti/Tv) ratio to assess the variant quality and percentage of duplication. The mean coverage per sample was 70X, the average percentage of target bases covered at 30X was 74.8%, the average Ti/Tv ratio was 2.45 and the percentage of duplication was lower than 10%

Eight heterozygous variants in three genes were classified as ’Pathogenic’ and are listed in Table 2. Among them, we identified three concurrent *SCN9A* variants (c.2971G>T, c.2794A>C and c.5756A>G) in two patients from both cohorts; these subjects had classical SFN, exhibiting burning pain in the lower legs. Beyond VGSC variants, we identified *WFS1* variants, that have been previously reported to alter protein function in peripheral neuropathy (PN) patients with diabetes mellitus [14,15,16]. *WFS1* variants were seen in ITA42 and NET11 individuals; only NET11 had glucose intolerance.

Fifteen heterozygous variants in twelve genes were classified as Likely Pathogenic (LP) and are reported in Table 3. Among them, two variants map to VGSC genes (*SCN9A*: c.4612T>C and *SCN10A*: c.3674T>C), whereas three variants map to *ATP7B* gene. Notably, in individual ITA04, two LP variants (*ATP7B*: c.1993A>G and *COL6A2*: c.1572+1G>A) were reported; we observed that these variants co-segregated in their relatives diagnosed with SFN and distal myopathy. Additionally, two genetic variants in the *LZTR1* gene, previously linked to schwannomatosis, were observed. Among the 12 mutated genes listed in Table 3, only 4 (*ATL1*, *PMP22*, *SCN9A*, and *SCN10A*) have been previously associated with SFN.

Variants in genes associated with inherited peripheral neuropathies (IPN), characterised by distinct genetic inheritance patterns like Charcot–Marie–Tooth Disease, were also examined. These variants, categorised as either LP or Variants of Uncertain Significance (VUS), are detailed in Table 4. In particular, *KIF1A* and *NAGLU* were frequently selected, especially in patients experiencing symptoms such as burning pain and sheet intolerance.

**Figure 2:**
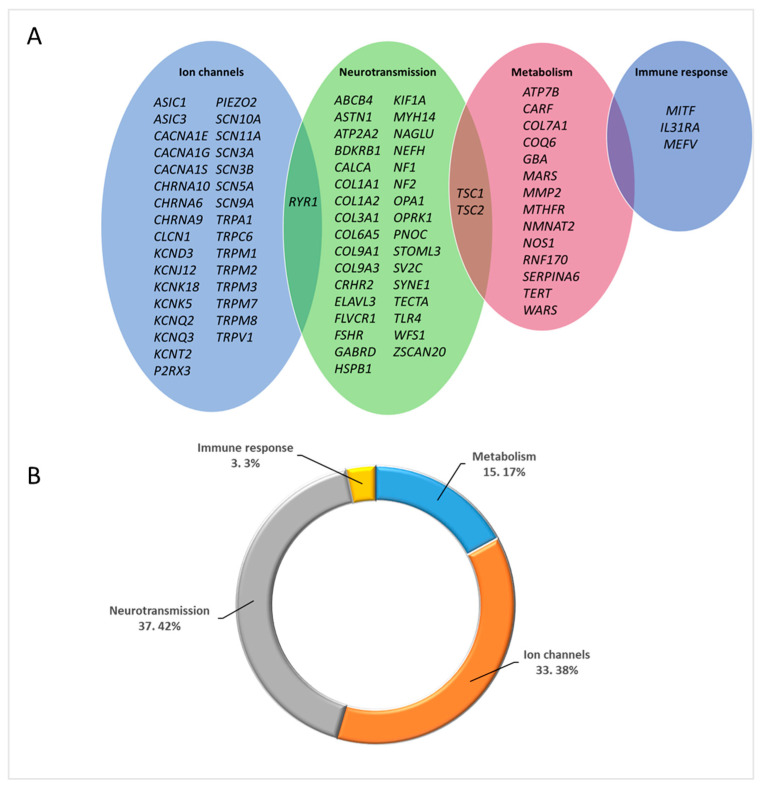
(**A**) Venn diagram of the 86 genes with VUS variants, categorised into four groups; ‘Ion channels’, ‘Neurotransmission’, ‘Metabolism’ and ‘Immune response’ adopted from Calvo, M; et al. [9]. (**B**) Percentage distribution of genes with distinct functions.

In the present study, we found 119 variants in heterozygous state that were categorized as VUS in 86 genes, as listed in Table S2. To better understand the functional biological systems they belong to, we categorized these genes into four groups: ‘Ion channels’, ‘Neurotransmission’, ‘Metabolism’, and ‘Immune response’, based on Calvo, M et al., [9]. We observed that the majority of genes with VUS variants were found in the ‘Neurotransmission’ group, followed by the ‘Ion channels’ category, as shown in Figure 2.

### 2.3. Summary of Prioritized Variants

To summarize, VGSC gene variants were observed in 23.8% of subjects, while TRP channel gene variants were seen in 19.3% of cases; 6.81% of subjects had no prioritized variants. Table 5 displays the top 10 genes with the highest sample count of prioritized variants in the analysed cohort, categorised based on their observed prevalence in individuals. It is important to note that each occurrence of the same variant has been counted separately when present in different individuals.

### 2.4. Pathway Analysis

To evaluate the enrichment in specific biological processes, a pathway analysis was conducted separately in cases and healthy controls using the WebGestalt tool and the PANTHER database as displayed in Figure 1B [49,50]. The analysis was performed without applying a gene panel filter, to avoid bias related to the pre-selection of painful SFN genes. After the application of the variant filtering pipelines explained in the Methods section, we selected 4254 genes in cases and 1226 genes in controls. The WebGestalt tool selects unambiguously annotated genes from the chosen database, which reduces the number of input genes available for pathway analysis; for this reason, only 589 genes for cases and 191 genes for controls were considered as input for pathway analysis using the PANTHER database. The top five pathways that emerged from cases and controls are described in Table 6A,B, respectively.

The ‘Nicotinic Acetylcholine Receptor Signaling’ (NARS) pathway was the only one significantly enriched in cases (FDR < 0.10 and *p* < 0.001); no pathways were significantly enriched in healthy control. The NARS pathway includes 93 genes; 52 out of the 589 input genes were part of this pathway. When checking the variants mapping to the 52 genes, we observed two variants in *CHRNA6* and *CACNA1S* that were found to be causative and classified as VUS.

## 3. Discussion

We applied WES to identify genetic variants and genes involved in painful SFN, which mainly affects individuals with age of onset around 50 [1]. In the present study, we focused on well-characterised cohorts of patients with early SFN onset (≤45 years) to increase the likelihood of finding variants involved in the genetic predisposition to painful SFN. We included samples from two distinct European origins (Italy and The Netherlands) who share comparable clinical features. To prioritize variants involved in pain, we manually curated a gene panel encompassing all the phenotypes and the clinical symptoms associated with painful SFN.

The cohort analysis unveiled two subjects harboring identical combinations of *SCN9A* genetic variants, as illustrated in Table 2. Moreover, a consistent trend emerged across both European cohorts; in fact, we observed that some of the genes mutated in one cohort were also mutated in the other cohort. Detailed discussion on the genetic variants observed in VGSC genes, *WFS1*, *COL7A1*, *ATP7B*, and *TRPA1* genes are provided subsequently.

### 3.1. Voltage-Gated Sodium Channel Variants

In the analysed cohorts, two individuals had three identical SCN9A variants. Two of these variants, *SCN9A*: c.2971G>T and *SCN9A*: c.2794A>C, were found to be in cis with each other and were classified as ‘Pathogenic’ based on functional and genetic studies [7,51]; a study indicated that the combination of all three variants contributes to NP [18]. Among the *SCN9A* variants, three were newly identified, while the others have been previously reported. Three out of the four *SCN10A* variants were novel, as well as all the *SCN11A* variants. The *SCN10A*: c.41G>T was observed in two individuals and had been previously reported in four SFN patients; however the familial co-segregation study was inconclusive for this variant [7]. Our findings are in line with other genetic studies related to SFN, indicating that *SCN9A* variants are more prevalent than other VGSC variants; however, they do not fully explain the observed phenotype. It is important to note that variants in VGSC variants are widely distributed across the channel, implying that there is no specific hotspot region that can be pinpointed with certainty.

### 3.2. WFS1 Pathogenic Variants

The variants mapping to *WFS1* (Wolframin ER Transmembrane Glycoprotein) were classified as ‘Pathogenic’ and had been associated with neurodegenerative disorders, including non-classic WFS1 Spectrum Disorders (WSD). Non-classic WSD has autosomal dominant inheritance, causing neurological abnormalities, glucose intolerance, hearing loss, and psychiatric disorders [15,16]. According to the literature, autosomal recessive inheritance leads to the absence of protein formation, and conversely, autosomal dominant inheritance, leads to modification of the protein product, with the disruption of calcium homeostasis via the endoplasmic reticulum stress pathway and the axonal degeneration in small fibers [52]. Because calcium dyshomeostasis is also involved in SFN pathogenesis, the variants in *WFS1* were classified as ‘Pathogenic’ [53]. As reported in Table 2, we identified two heterozygous variants in ITA42 and NET11 individuals carrying the *WFS1*:c.409_424dupGGCCGTCGCGAGGCTG, and *WFS1*:c.2648_2651delTCTT variants respectively. The variant identified in ITA42 individuals causes a complete loss of the WFS1 protein due to an early stop signal, which is associated with recessive WSD and type II diabetes mellitus with neurodegeneration. The variant detected in NET11 individuals, who exhibit glucose intolerance alongside SFN, is known to cause a complete breakdown of the WFS1 protein. According to De Heredia et al., both the reported variants fall under genotypic class A, which means that no protein product is generated [15]. Due to haploinsufficiency, these variants may also be causative in the dominant mode of inheritance. Based on these data, we have classified these two variants as ‘Pathogenic’ in our cohort.

### 3.3. COL7A1 Likely Pathogenic Variants

Among the LP category, we have identified two variants in *COL7A1* (Collagen Type VII Alpha 1 Chain). The reported variants (Table 3) have been previously linked to various forms of ‘Epidermolysis bullosa’ (EB) and can be inherited in both dominant and recessive modes. This particular gene was chosen because its variants exhibit phenotypes other than EB, including NP and sensory impairment. Recently, individuals with EB have been observed with a significant decrease in IENFD, which is also linked with SFN [54]. In ITA34 individuals, we observed a frameshift variant (*COL7A1*:c.497dupA), which has been previously reported in both the dominant and recessive forms of EB [33,34]. Another variant (*COL7A1*: c.4373C>T) was found in NET33 subjects, who had both itch (known symptom of EB) and painful SFN. This variant was previously found in patients with EB in dominant form; however, it was not the direct cause of EB. This leads us to question whether this variant is more closely related to SFN rather than EB [55]. Schmidt et al. found that mutations in the *COL7A1* gene in EB patients reduced IEFND and may lead to SFN [54]. As it has not been previously associated with SFN, we have classified *COL7A1* variants as LP.

### 3.4. ATP7B Genetic Variants

As part of this study, we identified six heterozygous variants in the *ATP7B* (ATPase Copper Transporting Beta) gene as shown in Figure S1. This gene is linked to Wilson’s disease in its recessive form and is associated with intellectual disability, enteropathy, deafness, peripheral neuropathy, ichthyosis and keratodermia (MEDNIK) syndrome in its dominant form [56,57]. Wilson’s disease is characterised by copper build-up and symptoms including liver malfunction and neurological abnormalities [56]. The *ATP7B* gene is also associated with SFN, and demyelinating polyneuropathy may occur with moderate Wilson’s disease symptoms [58,59]. In the case of MEDNIK syndrome, it is known that it may present with both PN and itch. We hypothesize that mutations in the *ATP7B* gene may cause these symptoms through disruptions in the AP1 protein complex, which is responsible for protein sorting, impacting the intracellular trafficking of ATP7B copper pumps. Indeed, mutations in the associated *AP1S1* (Adaptor Related Protein Complex 1 Subunit Sigma 1) gene can impact copper metabolism, contributing to MEDNIK syndrome. Disruptions in copper levels may affect ATP7B polarity, leading to abnormal copper metabolism and the manifestation of MEDNIK syndrome. All the reported variants in this gene either affect the protein folding or disrupt the copper transportation. Although *ATP7B* variants are typically associated with an autosomal recessive inheritance pattern, the *ATP7B*:c.3207C>A heterozygous variant has been previously reported in individuals with sensory disturbances; it has been speculated that subjects with this variant have a late-onset of the disease and neurological defects [39]. Additionally, the ATP7B:c.1993A>G variant has been confirmed to follow an autosomal dominant inheritance pattern, as it segregated with the disease in affected members of a family in Table 3.

### 3.5. Enrichment of TRPA1 Variants

We identified five *TRPA1* genetic variants in six individuals in the VUS category, as shown in Figure S2. TRPA1 contains a long ankyrin repeat domain at the N-terminus, which is a known hotspot region of this gene [60]. Mutations in *TRPA1*, particularly in the ankyrin repeat domain, sensitize the channel and lead to the induction of pain [61]. Additionally, genetic variants in this domain are recognized for their activation by cold temperatures [60]. In this study, two patients, ITA28 and ITA50, experienced cold allodynia, while another two, NET19 and NET31, who have the same variant, showed sheet intolerance. All identified *TRPA1* variants were located in the hotspot region of this channel. The genetic pathophysiology of SFN and pain is likely influenced by variants located in this hotspot region of TRPA1. Due to a lack of functional study on these variants, we classified them as VUS.

### 3.6. NARS Pathway

To better understand the functional role of genes involved in the phenotype of interest, a pathway analysis was performed. The analysis revealed the NARS pathway as the only significant one. It consists of 13 components, including Nicotinic Acetylcholine Receptors (NARs) and calcium ion channels. NARs are ligand-gated ion channels, formed by pentameric protein subunits [62], that play a crucial role in rapid signal transmission at synapses [63]. Different subunit combinations form NAR subtypes, which are present in different regions of the nervous system [64]. In dorsal root ganglia (DRG), NARs are involved in pain transmission by assisting in neuronal excitability [65]. Mutations in genes encoding NAR subunits can affect pain transmission. In our cohorts, rare VUS variants were found in *CHRNA6* and *CACNA1S* genes, which are part of the NARS pathway. As regards the *CHRNA6* gene, it is known that loss-of-function variants in this gene increase allodynia, whereas gain-of-function mutations decrease pain. It was also reported that the expression of the α6β4 (encodes for *CHRNA6* gene) subtype desensitizes the P2X2/3 receptors, which inhibits them and diminishes pain [66]. Variants found in the *CACNA1S* gene are associated with myalgic myopathy, linking it with pain transmission [67].

The variants discovered in this study will offer valuable insights into personalized treatments for pain relief. Indeed, NARs are potential therapeutic targets for pain, while calcium ion channels are already established pain targets [64].

## 4. Materials and Methods

### 4.1. Cohort Characterisation

From 2014 to 2021, subjects with SFN of non-Finnish Caucasian European descent were recruited at Maastricht University Medical Center+ (MUMC+), Maastricht, The Netherlands (n = 36), and Fondazione I.R.C.C.S. Istituto Neurologico Carlo Besta (FINCB), Milan, Italy (n = 52). In the Netherlands, MUMC+ is a tertiary referral hospital for SFN and other neurological conditions, while FINCB is a national referral center for numerous neurological diseases, including SFN. All the subjects were ≤45 years old at disease onset, allowing us to study an Early-Onset (EO) SFN cohort. Forty Caucasian Italian unrelated healthy controls (HC) were also recruited in IRCCS San Raffaele Hospital (HSR), Milan-Italy (n = 40). At the time of sampling, the HC samples had an average of 50 years with a median of 43 years of age. Medical history and clinical data were collected and recorded for all the subjects [51]. SFN diagnosis was carried out based on evidence of reduced IENFD in skin biopsies and/or abnormal temperature threshold testing (TTT) without signs of large nerve fiber involvement in nerve conduction study (NCS) [2]. The study was performed under the approval of the Ethical Committee of the involved clinical centers and all the recruited subjects signed the informed consent. In Figure 1, a summary of the involved individuals and the workflow of the study is depicted.

### 4.2. DNA Extraction

Genomic DNA was extracted from whole blood or saliva samples using a NucleoSpin8 Blood Isolation kit (Macherey-Nagel, Düren, Germany) or a QIAamp DNA Blood Maxi Kit, Puregene^®^ Blood Core Kit l (Qiagen, Hilden, Germany). DNA isolation was performed according to the manufacturer’s instructions and stored at −20 °C. Quality and quantity checks of extracted DNA were performed using agarose gel, a Qubit 2.0 Fluorometer (Thermo Fisher, San Jone, CA, USA), and a Nanodrop Spectrophotometer (Thermo Fisher, San Jone, CA, USA).

### 4.3. Whole-Exome Sequencing

Libraries were prepared using the SureSelect Human All Exon QXT v4/v5/v7 kit [68] according to the manufacturer’s protocols. The size distribution of enriched DNA was checked using the 2100 Bioanalyzer [68]. Samples were sequenced using the HiSeq 2000/2500 and run in pair-ended mode (2 × 101 or 2 × 150) [69].

### 4.4. Variant Filtration and Classification

Reads were processed and aligned to the reference hg19 human genome using an in-house bioinformatics pipeline; variants were identified and filtered based on multiple criteria including depth, impact on protein product, and absence in internal databases, population frequency, conservation, pathogenicity prediction, and phenotype-driven gene panel. Exonic and splice site variants (+/−10 bp) meeting the following criteria were selected for further analysis: read number ≥ 10; alternative variant call ≥ 25%; variation reads or allelic depth ≥ 6; QUAL score ≥ 100. The variants were filtered based on the Variant Allele Frequency (VAF). We considered rare variants with a Minor Allele Frequency (MAF) of less than 5% in population databases such as dbSNP and GnomAD non-Finnish European (NFE) for further analyses. Additionally, we selected the variants that were absent in the HC cohort. A manually curated phenotype-driven gene panel of 592 genes (Table S1) was created and applied as a filter to increase specificity. Variant classification was performed according to the practice guidelines of the Association for Clinical Genetic Science (ACGS) and American College of Medical Genetics (ACMG) guidelines 2015 [70], as reported in Figure 1A. The Alamut Visual (Interactive Biosoftware, Rouen, France) software was used and only variants classified as ‘Pathogenic’, LP, and VUS, were retained. We grouped the genes containing the qualifying variants classified as VUS into four categories, adapted from Calvo, M; et al. [9]: “Ion channel,” “Neurotransmission,” “Metabolism,” and “Immune response.”

### 4.5. Phenotype-Driven Gene Panel Creation

A pain-related gene panel composed of 592 genes has been created for the purpose of this study (Table S1). Genes were selected for a possible role in pain, applying the following criteria (i) genes reported in the literature as being associated with pain, (ii) genes present in genetic pain databases (International Association for Study of Pain database and Human Pain Genetics database), (iii) genes reported in the Human Phenotype Ontology database and Online Mendelian Inheritance in Man (OMIM) as being associated with symptoms typical for peripheral neuropathy, neuropathic pain and associated symptoms such as pain hypersensitivity, paraesthesia, allodynia, restless legs, sicca syndrome, abnormal warm and cold sensation, hypohidrosis, hyperhidrosis, hyperalgesia, neuropathic itch, thermal sensory loss, pinprick loss, impotence, hot flushes, orthostatic dizziness, cardiac palpitations, bowel disturbances (constipation, diarrhoea, irritability, gastroparesis, cramps), dry eyes, dry mouth, burning pain, shooting pain, stocking pattern of sensory loss and micturition disturbances.

### 4.6. Pathway Analysis

Pathway analysis was conducted using the WebGestalt tool with over-representation analysis. The Benjamin–Hochberg method was used to control for false discovery rates [49]. Input data included “qualifying genes” obtained when applying variant filters without selecting for genes related to pain both in cases (EO-SFN patients) and controls, mapped to the PANTHER database [50]. Duplicated genes were removed, and LoFtool scores were used to filter genes [71]. The case (FINCB and MUMC+) and control (HSR) gene lists were analysed separately in WebGestalt, associating them with biological pathways as shown in Figure 1B.

## 5. Conclusions

This study contributes to elucidating the complex genetic nature of painful SFN in EO individuals for whom we expect a genetic predisposition leading to the early onset phenotype. The WES approach proved to be effective in detecting causative variants and establishing genotype-phenotype relationships [72]. The compiled gene list consists of 93 genes prioritized as ‘Pathogenic’, LP, and VUS; we propose that this list may function as an initial screening tool in genetic investigations. However, validation of this gene list in larger cohorts with diverse ethnicities is essential.

**Figure 3:**
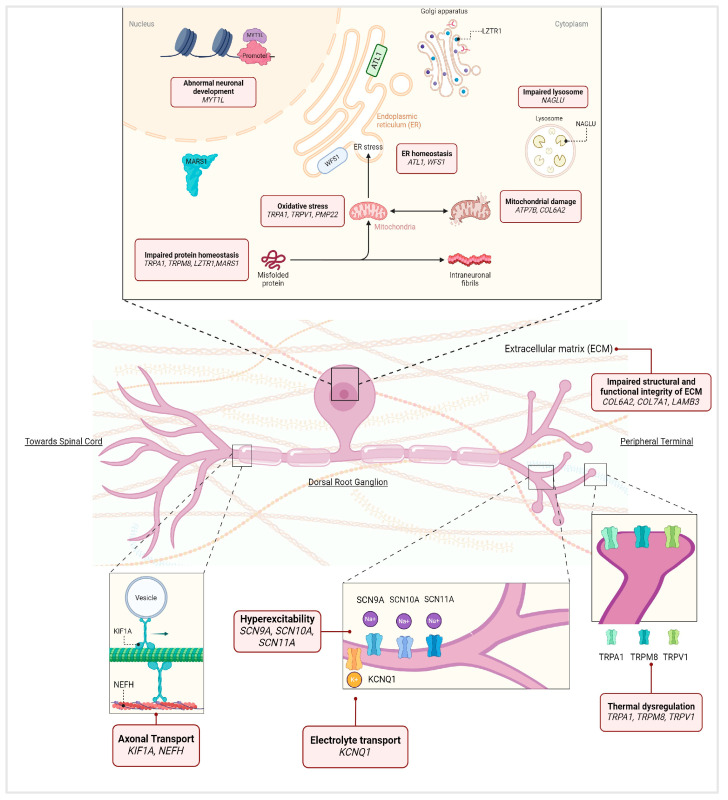
This figure summarizes the genes listed in Table 2, Table 3, Table 4 and Table 5, emphasizing their roles in dorsal root ganglion (DRG) neurons. In particular, it includes pathogenic variants in *SCN9A*, *SCN11A*, and *WFS1* (previously reported in Table 2); likely pathogenic (LP) variants in *ATL1*, *ATP7B*, *COL6A2*, *COL7A1*, *KCNQ1*, *LAMB3*, *LZTR1*, *MYT1L*, *PMP22*, and *SCN10A* (Table 3); variants in inherited peripheral neuropathy-related genes *ATL1*, *KIF1A*, *MARS1*, *NAGLU*, *NEFH*, *PMP22*, and *SCN9A* (Table 4); the top 10 genes with the highest sample count which are *SCN9A*, *TRPA1*, *ATP7B*, *SCN10A*, *COL7A1*, *WFS1*, *NAGLU*, *SCN11A*, *TRPM8*, and *TRPV1* (Table 5).

The genetic variants selected by our study were absent in HC; their corresponding genes implicated different functional processes, including VGSC genes, TRP genes, and copper metabolism-related genes, which suggest their involvement in disease initiation and pain regulation as shown in Figure 3. Pathway analysis revealed that there is an enrichment in the NARS pathway in patients compared to healthy controls; based on this data we can speculate on the disruption of the synaptic transmission mechanism as a probable pathogenic mechanism participating in disease manifestation. This study highlights the clinical relevance of WES, emphasizing its capacity to advance diagnosis and potentially identify treatment targets for a clinically heterogeneous condition like SFN. The results potentially lay the groundwork for genetic screening initiatives and may catalyze new functional studies into genes such as *COL7A1* and *TRPA1*. These studies could have broader implications, influencing therapeutic development for patients affected not only by SFN but also by various forms of painful PN.

This figure illustrates the potential biological functions of each gene and the impact of mutations on these processes as reported in the literature [7,26,37,54,73,74,75,76,77,78,79,80] (adapted from Lischka, A. et al. and Hardiman, O. et al. [81,82]).

## Figures and Tables

**Table 1:** Summary of the analysed cohort.

	FINCB ^1^	MUMC+ ^2^	HC ^3^	Total
Individuals (n)	52	36	40	88
Gender (F)	29	27	27	56
Average age at onset	35	29	-	33.3
IENFD (↓)	52	22	-	74
TTT (Abnormal)	-	24	-	24

^1^ FINCB: Fondazione I.R.C.C.S. Istituto Neurologico Carlo Besta, Italy; ^2^ MUMC+: Maastricht University Medical Center+, Netherlands; ^3^ HC: healthy control, Italy.

**Table 2:** Pathogenic variants identified in EO-SFN patients.

Individuals	Gene Name	c.change	p.change	Transcript	MAF gnomAD NFE	VAF (%)	CADD Scores	References
ET14, NET22	*SCN9A*	c.684C>G	p.Ile228Met	NM_001365536.1	0.00159	46.3, 42.5	20.2	[7,17]
NET12	*SCN9A*	c.2567G>A	p.Gly856Asp	NM_002977.3	−1	48.5	24.6	[7,18]
ITA03, NET36	*SCN9A*	c.2794A>C	p.Met932Leu	NM_002977.3	0.0028	27.2, 45.1	20.1	[7,18]
		c.2971G>T	p.Val991Leu	NM_002977.3	0.0028	47.3, 41.3	9.81	
		c.5723A>G	p.Asp1908Gly	NM_002977.3	0.0033	46.2, 58.3	22.8	
NET17	*SCN11A*	c.1744G>A	p.Ala582Thr	NM_001349253.1	0.000264	49.5	19.4	[7,19]
ITA42	*WFS1*	c.409_424dupGGCCGTCGCGAGGCTG	p.Val142fs	NM_006005.3	0.000056	50	NA	[14,15,16]
NET11	*WFS1*	c.2648_2651delTCTT	p.Phe883SerfsTer68	NM_006005.3	0.000195	60.4	NA	[16,20]

Individuals from the Netherlands cohort are tagged using the NET prefix, while individuals from Italy have the ITA prefix; c.change: location in coding DNA (cDNA); p.change: location in protein; MAF gnomAD NFE: Minor Allele Frequency Genome Aggregation Database for non-Finnish Eu-ropean population; VAF: Variant Allele Frequency of that individual (depth of alternate allele/total depth at that positionx 100), Combined Annotation Dependent Depletion (CADD) scores.

**Table 3:** Likely Pathogenic variants identified in EO-SFN patients.

Individuals	Gene Name	c.change	p.change	Transcript	MAF gnomAD NFE	VAF (%)	CADD Scores	References
ITA03	*SCN9A*	c.4612T>C	p.Trp1538Arg	NM_002977.3	0.002461	50	18.64	[7]
ITA04	*ATP7B*	c.1993A>G	p.Met665Val	NM_000053.3	0.0004051	48.5	22	[21]
	*COL6A2*	c.1572+1G>A	-	NM_001849.4	−1	50	33	[22,23]
ITA17	*PMP22*	c.88G>A	p.Val30Met	NM_000304.4	0.00001549	50.7	22.8	[24,25]
ITA19	*ATL1*	c.1247G>A	p.Arg416His	NM_015915.4	0.00006481	47.2	24.7	[26,27]
ITA26	*LZTR1*	c.1084C>T	p.Arg362 *	NM_006767.4	0.00006208	47.6	37	[28,29]
ITA27	*MYT1L*	c.1672C>T	p.Arg558Cys	NM_001303052.1	−1	52.3	32	[30,31]
ITA34	*COL7A1*	c.497dupA	p.Val168fs	NM_000094.3	0.00006164	39.2	NA	[32,33,34]
ITA45	*SCN10A*	c.3674T>C	p.Ile1225Thr	NM_006514.3	0.0008442	46.1	26.7	[7]
ITA46	*ATP7B*	c.2138A>G	p.Tyr713Cys	NM_000053.3	0.00002337	48	26.5	[21,35]
NET04	*LZTR1*	c.2066C>G	p.Ser689Cys	NM_006767.4	−1	69.1	22.2	[36,37]
NET19	*KCNQ1*	c.590C>T	p.Pro197Leu	NM_000218.2	0.000115	49.1	29.3	[38]
NET23	*ATP7B*	c.3207C>A	p.His1069Gln	NM_000053.3	0.00000883	51.1	23	[39]
**NET30**	***LAMB3***	**c.1903C>T**	p.Arg635 *	**NM_000228.3**	**0.00103**	**52.6**	**36**	**[40]**
NET33	*COL7A1*	c.4373C>T	p.Pro1458Leu	NM_000094.3	0.003635	72.2	27.7	[41]

* Individuals from the Netherlands cohort are tagged as NET and ITA for Italy; c.change: location in cDNA; p.change: location in protein; MAF gnomAD NFE: Minor Allele Frequency Genome Aggregation Database for non-Finnish European population; VAF: Variant Allele Frequency in that sample (depth of alternate allele/total depth at that position*100), Combined Annotation Dependent Depletion (CADD) scores, Bold characters indicate novel variants that are only reported in ClinVar database.

**Table 4:** Likely Pathogenic and Variants of Uncertain Significance identified in genes related to IPN.

Variants	Gene-Disease Association	Classification	References
*ATL1*(NM_015915.4): c.1247G>A	HSN ID	LP	[26,27]
***KIF1A*** **(NM_001244008.1): c.694G>A**	**HSN IIC**	**VUS**	**[42]**
***KIF1A*** **(NM_001244008.1): c.4334G>A**	**HSN IIC**	**VUS**	**[43]**
***MARS1*** **(NM_004990.4): c.1793G>A**	**CMTD, axonal, type 2U**	**VUS**	**[44]**
*NAGLU*(NM_000263.4): c.1000G>A	CMTD, axonal, type 2V	LP	[45,46]
***NAGLU*** **(NM_000263.4): c.384-10C>G**	**CMTD, axonal, type 2V**	**VUS**	**[47]**
***NAGLU*** **(NM_000263.4): c.527A>G**	**CMTD, axonal, type 2V**	**VUS**	**[48]**
***NEFH*** **(NM_021076.3): c.736C>G**	**CMTD, axonal, type 2CC**	**VUS**	**-**
*PMP22*(NM_000304.4): c.88G>A	HNPP	LP	[24,25]
*SCN9A*(NM_002977.3): c.4612T>C	HSAN IID, PEM, SFN	LP	[7]
*SCN9A*(NM_002977.3): c.3689T>C	HSAN IID, PEM, SFN	VUS	[7]

HSN = Hereditary Sensory Neuropathy; CMTD = Charcot–Marie–Tooth Disease; HNPP = Hereditary Neuropathy with Pressure Palsies; HSAN = Hereditary Sensory and Autonomic Neuropathy; LP = Likely Pathogenic; VUS = Variants of Uncertain Significance. Bold variants indicate novel variants that are only reported in the ClinVar database.

**Table 5:** List of top 10 genes with the highest sample count with relative prevalence.

Gene Name	Pathogenic	Likely Pathogenic	VUS	Total
*SCN9A*	9 ^§^	1	5	15
*TRPA1*	0	0	6 *	6
*ATP7B*	0	3	3	6
*SCN10A*	0	1	5 ^#^	6
*COL7A1*	0	2	2	4
*WFS1*	0	2	2	4
*NAGLU*	0	1	2	3
*SCN11A*	0	1	2	3
*TRPM8*	0	0	3	3
*TRPV1*	0	0	2	2

^§,^ *^, #^ denotes that the same variant has been counted as a separate variant when present in different individuals. ^§^ One patient was heterozygous for *SCN9A*:c.2794A>C, *SCN9A*:c.2971G>T, *SCN9A*:c.5723A>G, *SCN9A*:c.4612T>C; ^§^ One patient was heterozygous for *SCN9A*:c.2794A>C, *SCN9A*:c.2971G>T, and *SCN9A*:c.5723A>G; ^§^ Two patients were heterozygous for *SCN9A*:c.684C>G; * Two patients were heterozygous for *TRPA1*:c.2065A>G; ^#^ Two patients were heterozygous for *SCN10A*:c.41G>T.

**Table 6:** Pathway analysis results for cases (A) and healthy controls (B).

**A: Pathway Analysis Results from Cases**					
**Pathway**	**Size**	**Expect**	**Ratio**	***p* Value**	**FDR**
Nicotinic acetylcholine receptor signalling pathway	93	24.173	2.1511	3.7977 × 10^−10^	4.2914 × 10^−8^
Integrin signalling pathway	166	43.148	1.3210	0.0081741	0.33901
Blood coagulation	38	9.8773	1.7211	0.0091	0.33901
Alzheimer disease-presenilin pathway	112	29.112	1.3396	0.0212	0.49960
Cadherin signalling pathway	153	39.769	1.28245	0.0221	0.49960
**B: Pathway Analysis Results from Healthy Controls**					
**Pathway**	**Size**	**Expect**	**Ratio**	***p* value**	**FDR**
Cadherin signalling pathway	153	12.9	1.861	0.00159	0.179
JAK/STAT signalling pathway	15	1.26	3.955	0.00602	0.277
Nicotinic acetylcholine receptor signalling pathway	93	7.89	1.913	0.00943	0.277
Integrin signalling pathway	166	13.9	1.643	0.00983	0.277
Wnt signalling pathway	294	24.7	1.412	0.01726	0.390

Pathway: name of the pathway; Size: number of genes in the pathway; Expect: number of genes overlapped between the pathway and the given input; Ratio: enrichment ratio; FDR: False Discovery Rate.

## Data Availability

Data sharing is not applicable to this article.

## Supplementary Materials

The supporting information can be downloaded at: https://www.mdpi.com/article/10.3390/ijms25137248/s1.

## References

[1] Bitzi L.M., Lehnick D., Wilder-Smith E.P. (2021). Small Fiber Neuropathy: Swiss Cohort Characterization. Muscle Nerve.

[2] de Greef B.T.A., Hoeijmakers J.G.J., Gorissen-Brouwers C.M.L., Geerts M., Faber C.G., Merkies I.S.J. (2018). Associated Conditions in Small Fiber Neuropathy—A Large Cohort Study and Review of the Literature. Eur. J. Neurol..

[3] Sène D. (2018). Small Fiber Neuropathy: Diagnosis, Causes, and Treatment. Jt. Bone Spine.

[4] Verdugo R.J., Matamala J.M., Inui K., Kakigi R., Valls-Solé J., Hansson P., Nilsen K.B., Lombardi R., Lauria G., Petropoulos I.N. (2022). Review of Techniques Useful for the Assessment of Sensory Small Fiber Neuropathies: Report from an IFCN Expert Group. Clin. Neurophysiol..

[5] Devigili G., Cazzato D., Lauria G. (2020). Clinical Diagnosis and Management of Small Fiber Neuropathy: An Update on Best Practice. Expert Rev. Neurother..

[6] Raasing L.R.M., Vogels O.J.M., Veltkamp M., Van Swol C.F.P., Grutters J.C. (2021). Current View of Diagnosing Small Fiber Neuropathy. J. Neuromuscul. Dis..

[7] Eijkenboom I., Sopacua M., Hoeijmakers J.G.J., De Greef B.T.A., Lindsey P., Almomani R., Marchi M., Vanoevelen J., Smeets H.J.M., Waxman S.G. (2019). Yield of Peripheral Sodium Channels Gene Screening in Pure Small Fibre Neuropathy. J. Neurol. Neurosurg. Psychiatry.

[8] Ślęczkowska M., Almomani R., Marchi M., Salvi E., de Greef B.T.A., Sopacua M., Hoeijmakers J.G.J., Lindsey P., Waxman S.G., Lauria G. (2022). Peripheral Ion Channel Genes Screening in Painful Small Fiber Neuropathy. Int. J. Mol. Sci..

[9] Calvo M., Davies A.J., Hébert H.L., Weir G.A., Chesler E.J., Finnerup N.B., Levitt R.C., Smith B.H., Neely G.G., Costigan M. (2019). The Genetics of Neuropathic Pain from Model Organisms to Clinical Application. Neuron.

[10] Ślęczkowska M., Misra K., Santoro S., Gerrits M.M., Hoeijmakers J.G.J. (2023). Ion Channel Genes in Painful Neuropathies. Biomedicines.

[11] Marchi M., Salvi E., Andelic M., Mehmeti E., D’Amato I., Cazzato D., Chiappori F., Lombardi R., Cartelli D., Devigili G. (2022). TRPA1 Rare Variants in Chronic Neuropathic and Nociplastic Pain Patients. Pain.

[12] Martinelli-Boneschi F., Colombi M., Castori M., Devigili G., Eleopra R., Malik R.A., Ritelli M., Zoppi N., Dordoni C., Sorosina M. (2017). COL6A5 Variants in Familial Neuropathic Chronic Itch. Brain.

[13] Sainio M.T., Aaltio J., Hyttinen V., Kortelainen M., Ojanen S., Paetau A., Tienari P., Ylikallio E., Auranen M., Tyynismaa H. (2022). Effectiveness of Clinical Exome Sequencing in Adult Patients with Difficult-to-Diagnose Neurological Disorders. Acta Neurol. Scand..

[14] Inoue H., Tanizawa Y., Wasson J., Behn P., Kalidas K., Bernal-Mizrachi E., Mueckler M., Marshall H., Donis-Keller H., Crock P. (1998). A Gene Encoding a Transmembrane Protein Is Mutated in Patients with Diabetes Mellitus and Optic Atrophy (Wolfram Syndrome). Nat. Genet..

[15] De Heredia M.L., Clèries R., Nunes V. (2013). Genotypic Classification of Patients with Wolfram Syndrome: Insights into the Natural History of the Disease and Correlation with Phenotype. Genet. Med..

[16] Astuti D., Sabir A., Fulton P., Zatyka M., Williams D., Hardy C., Milan G., Favaretto F., Yu-Wai-Man P., Rohayem J. (2017). Monogenic Diabetes Syndromes: Locus-specific Databases for Alström, Wolfram, and Thiamine-responsive Megaloblastic Anemia. Hum. Mutat..

[17] Eijkenboom I., Sopacua M., Otten A.B.C., Gerrits M.M., Hoeijmakers J.G.J., Waxman S.G., Lombardi R., Lauria G., Merkies I.S.J., Smeets H.J.M. (2019). Expression of Pathogenic SCN9A Mutations in the Zebrafish: A Model to Study Small-Fiber Neuropathy. Exp. Neurol..

[18] Zeberg H., Dannemann M., Sahlholm K., Tsuo K., Maricic T., Wiebe V., Hevers W., Robinson H.P.C., Kelso J., Pääbo S. (2020). A Neanderthal Sodium Channel Increases Pain Sensitivity in Present-Day Humans. Curr. Biol..

[19] Zhou X., Xiao Z., Xu Y., Zhang Y., Tang D., Wu X., Tang C., Chen M., Shi X., Chen P. (2017). Electrophysiological and Pharmacological Analyses of Nav1.9 Voltage-Gated Sodium Channel by Establishing a Heterologous Expression System. Front. Pharmacol..

[20] Rohayem J., Ehlers C., Wiedemann B., Holl R., Oexle K., Kordonouri O., Salzano G., Meissner T., Burger W., Schober E. (2011). Diabetes and Neurodegeneration in Wolfram Syndrome: A Multicenter Study of Phenotype and Genotype. Diabetes Care.

[21] Martinelli D., Dionisi-Vici C. (2014). AP1S1 Defect Causing MEDNIK Syndrome: A New Adaptinopathy Associated with Defective Copper Metabolism. Ann. N. Y. Acad. Sci..

[22] National Center of Biotechnology Information NM_001849.4(COL6A2):C.1572+1G>A AND Not Provided—ClinVar—NCBI. https://www.ncbi.nlm.nih.gov/clinvar/RCV000423788/.

[23] Inoue M., Saito Y., Yonekawa T., Ogawa M., Iida A., Nishino I., Noguchi S. (2021). Causative Variant Profile of Collagen VI-Related Dystrophy in Japan. Orphanet J. Rare Dis..

[24] National Center for Biotechnology Information NM_000304.4(PMP22):C.88G>A (p.Val30Met) AND Charcot-Marie-Tooth Disease, Type I—ClinVar—NCBI. https://www.ncbi.nlm.nih.gov/clinvar/RCV000796876/.

[25] Shames I., Fraser A., Colby J., Orfali W., Snipes G.J. (2003). Phenotypic Differences between Peripheral Myelin Protein-22 (PMP22) and Myelin Protein Zero (P0) Mutations Associated with Charcot-Marie-Tooth-Related Diseases. J. Neuropathol. Exp. Neurol..

[26] Xiao X.W., Du J., Jiao B., Liao X.X., Zhou L., Liu X.X., Yuan Z.H., Guo L.N., Wang X., Shen L. (2019). Novel ATL1 Mutation in a Chinese Family with Hereditary Spastic Paraplegia: A Case Report and Review of Literature. World J. Clin. Cases.

[27] Guelly C., Zhu P.P., Leonardis L., Papić L., Zidar J., Schabhüttl M., Strohmaier H., Weis J., Strom T.M., Baets J. (2011). Targeted High-Throughput Sequencing Identifies Mutations in Atlastin-1 as a Cause of Hereditary Sensory Neuropathy Type I. Am. J. Hum. Genet..

[28] National Center for Biotechnology Information NM_006767.4(LZTR1):C.1084C>T (p.Arg362Ter) AND Not Provided—ClinVar—NCBI. https://www.ncbi.nlm.nih.gov/clinvar/RCV000329167/.

[29] Jordan J.T., Smith M.J., Walker J.A., Erdin S., Talkowski M.E., Merker V.L., Ramesh V., Cai W., Harris G.J., Bredella M.A. (2018). Pain Correlates with Germline Mutation in Schwannomatosis. Medicine.

[30] National Center for Biotechnology Information NM_001303052.2(MYT1L):C.1672C>T (p.Arg558Cys) AND Intellectual Disability, Autosomal Dominant 39—ClinVar—NCBI. https://www.ncbi.nlm.nih.gov/clinvar/RCV001253116/.

[31] D’Agnelli S., Arendt-Nielsen L., Gerra M.C., Zatorri K., Boggiani L., Baciarello M., Bignami E. (2019). Fibromyalgia: Genetics and Epigenetics Insights May Provide the Basis for the Development of Diagnostic Biomarkers. Mol. Pain.

[32] National Center for Biotechnology Information VCV000279784.32—ClinVar—NCBI. https://www.ncbi.nlm.nih.gov/clinvar/variation/279784/?oq=%22NM_000094.4(COL7A1):c.497dup(p.Val168fs)%22%5Bvarname%5D&m=NM_000094.4(COL7A1):c.497dup(p.Val168fs).

[33] Christiano A.M., D’Alessio M., Paradisi M., Angelo C., Mazzanti C., Puddu P., Uitto J. (1996). A Common Insertion Mutation in COL7A1 in Two Italian Families with Recessive Dystrophic Epidermolysis Bullosa. J. Investig. Dermatol..

[34] Varki R., Sadowski S., Uitto J., Pfendner E. (2007). Epidermolysis Bullosa. II. Type VII Collagen Mutations and Phenotype–Genotype Correlations in the Dystrophic Subtypes. J. Med. Genet..

[35] Loudianos G., Dessì V., Angius A., Lovicu M., Loi A., Deiana M., Akar N., Vajro P., Figus A., Cao A. (1996). Wilson Disease Mutations Associated with Uncommon Haplotypes in Mediterranean Patients. Hum. Genet..

[36] National Center for Biotechnology Information VCV002195419.1—ClinVar—NCBI. https://www.ncbi.nlm.nih.gov/clinvar/variation/2195419/.

[37] Farschtschi S.C., Mainka T., Glatzel M., Hannekum A.L., Hauck M., Gelderblom M., Hagel C., Friedrich R.E., Schuhmann M.U., Schulz A. (2020). C-Fiber Loss as a Possible Cause of Neuropathic Pain in Schwannomatosis. Int. J. Mol. Sci..

[38] Vanoye C.G., Desai R.R., Fabre K.L., Gallagher S.L., Potet F., DeKeyser J.M., Macaya D., Meiler J., Sanders C.R., George A.L. (2018). High Throughput Functional Evaluation of KCNQ1 Decrypts Variants of Unknown Significance. Circ. Genom. Precis. Med..

[39] Machado A.A.C., Deguti M.M., Genschel J., Cançado E.L.R., Bochow B., Schmidt H., Barbosa E.R. (2008). Neurological Manifestations and ATP7B Mutations in Wilson’s Disease. Parkinsonism Relat. Disord..

[40] National Center of Biotechnology Information NM_000228.3(LAMB3):C.1903C>T (p.Arg635Ter) AND Not Provided—ClinVar—NCBI. https://www.ncbi.nlm.nih.gov/clinvar/RCV000255105/.

[41] Yuan J.H., Schulman B.R., Effraim P.R., Sulayman D.H., Jacobs D.S., Waxman S.G. (2020). Genomic Analysis of 21 Patients with Corneal Neuralgia after Refractive Surgery. Pain Rep..

[42] National Center for Biotechnology Information VCV001063557.6—ClinVar—NCBI. https://www.ncbi.nlm.nih.gov/clinvar/variation/1063557/#id_second.

[43] National Center for Biotechnology Information VCV001384008.5—ClinVar—NCBI. https://www.ncbi.nlm.nih.gov/clinvar/variation/1384008/#id_second.

[44] National Center for Biotechnology Information NM_004990.4(MARS1):C.1793G>A (p.Arg598His) AND Severe Early-Onset Pulmonary Alveolar Proteinosis Due to MARS Deficiency—ClinVar—NCBI. https://www.ncbi.nlm.nih.gov/clinvar/RCV001647326/.

[45] National Center for Biotechnology Information NM_000263.4(NAGLU):C.1000G>A (p.Val334Ile) AND Not Provided—ClinVar—NCBI. https://www.ncbi.nlm.nih.gov/clinvar/RCV000761953/.

[46] Weber B., Guo X.H., Kleijer W.J., Van De Kamp J.J.P., Poorthuis B.J.H.M., Hopwood J.J. (1999). Sanfilippo Type B Syndrome (Mucopolysaccharidosis III B): Allelic Heterogeneity Corresponds to the Wide Spectrum of Clinical Phenotypes. Eur. J. Hum. Genet..

[47] National Center for Biotechnology Information NM_000263.4(NAGLU):C.384-10C>G AND Not Provided—ClinVar—NCBI. https://www.ncbi.nlm.nih.gov/clinvar/RCV001754503/.

[48] National Center for Biotechnology Information NM_000263.4(NAGLU):C.527A>G (p.Gln176Arg) AND Multiple Conditions—ClinVar—NCBI. https://www.ncbi.nlm.nih.gov/clinvar/RCV002595192/.

[49] Liao Y., Wang J., Jaehnig E.J., Shi Z., Zhang B. (2019). WebGestalt 2019: Gene Set Analysis Toolkit with Revamped UIs and APIs. Nucleic Acids Res..

[50] Mi H., Ebert D., Muruganujan A., Mills C., Albou L.P., Mushayamaha T., Thomas P.D. (2021). PANTHER Version 16: A Revised Family Classification, Tree-Based Classification Tool, Enhancer Regions and Extensive API. Nucleic Acids Res..

[51] Faber C.G., Hoeijmakers J.G.J., Ahn H.S., Cheng X., Han C., Choi J.S., Estacion M., Lauria G., Vanhoutte E.K., Gerrits M.M. (2012). Gain of Function Na V1.7 Mutations in Idiopathic Small Fiber Neuropathy. Ann. Neurol..

[52] Pallotta M.T., Tascini G., Crispoldi R., Orabona C., Mondanelli G., Grohmann U., Esposito S. (2019). Wolfram Syndrome, a Rare Neurodegenerative Disease: From Pathogenesis to Future Treatment Perspectives. J. Transl. Med..

[53] Gross F., Üçeyler N. (2020). Mechanisms of Small Nerve Fiber Pathology. Neurosci. Lett..

[54] Schmidt D., Díaz P., Muñoz D., Espinoza F., Nystrom A., Fuentes I., Ezquer M., Bennett D.L., Calvo M. (2022). Characterisation of the Pathophysiology of Neuropathy and Sensory Dysfunction in a Mouse Model of Recessive Dystrophic Epidermolysis Bullosa. Pain.

[55] Kern J.S., Kohlhase J., Bruckner-Tuderman L., Has C. (2006). Expanding the COL7A1 Mutation Database: Novel and Recurrent Mutations and Unusual Genotype-Phenotype Constellations in 41 Patients with Dystrophic Epidermolysis Bullosa. J. Investig. Dermatol..

[56] Bandmann O., Weiss K.H., Kaler S.G. (2015). Wilson’s Disease and Other Neurological Copper Disorders. Lancet. Neurol..

[57] Hartwig C., Zlatic S.A., Wallin M., Vrailas-Mortimer A., Fahrni C.J., Faundez V. (2019). Trafficking Mechanisms of P-Type ATPase Copper Transporters. Curr. Opin. Cell Biol..

[58] Sturniolo G.C., Lazzarini D., Bartolo O., Berton M., Leonardi A., Fregona I.A., Parrozzani R., Midena E. (2015). Small Fiber Peripheral Neuropathy in Wilson Disease: An In Vivo Documentation by Corneal Confocal Microscopy. Investig. Ophthalmol. Vis. Sci..

[59] Jung K.H., Ahn T.B., Jeon B.S. (2005). Wilson Disease With an Initial Manifestation of Polyneuropathy. Arch. Neurol..

[60] Moparthi L., Survery S., Kreir M., Simonsen C., Kjellbom P., Högestätt E.D., Johanson U., Zygmunt P.M. (2014). Human TRPA1 Is Intrinsically Cold- and Chemosensitive with and without Its N-Terminal Ankyrin Repeat Domain. Proc. Natl. Acad. Sci. USA.

[61] Meents J.E., Fischer M.J.M., McNaughton P.A. (2017). Sensitization of TRPA1 by Protein Kinase A. PLoS ONE.

[62] Stokes C., Treinin M., Papke R.L. (2015). Looking below the Surface of Nicotinic Acetylcholine Receptors. Trends Pharmacol. Sci..

[63] Orr-Urtreger A., Seldin M.F., Baldini A., Beaudet A.L. (1995). Cloning and Mapping of the Mouse Alpha 7-Neuronal Nicotinic Acetylcholine Receptor. Genomics.

[64] Hone A.J., McIntosh J.M. (2018). Nicotinic Acetylcholine Receptors in Neuropathic and Inflammatory Pain. FEBS Lett..

[65] Lang P.M., Burgstahler R., Sippel W., Irnich D., Schlotter-Weigel B., Grafe P. (2003). Characterization of Neuronal Nicotinic Acetylcholine Receptors in the Membrane of Unmyelinated Human C-Fiber Axons by in Vitro Studies. J. Neurophysiol..

[66] Wieskopf J.S., Mathur J., Limapichat W., Post M.R., Al-Qazzaz M., Sorge R.E., Martin L.J., Zaykin D.V., Smith S.B., Freitas K. (2015). The Nicotinic A6 Subunit Gene Determines Variability in Chronic Pain Sensitivity via Cross-Inhibition of P2X2/3 Receptors. Sci. Transl. Med..

[67] Periviita V., Palmio J., Jokela M., Hartikainen P., Vihola A., Rauramaa T., Udd B. (2023). CACNA1S Variant Associated with a Myalgic Myopathy Phenotype. Neurology.

[68] Agilent Technologies Agilent Technologies SureSelect QXT Target Enrichment for the Illumina Platform. https://www.agilent.com/cs/library/usermanuals/public/G9681-90000.pdf.

[69] Illumina Technologies HiSeq 2500 Specifications|Key Performance Parameters. https://www.illumina.com/systems/sequencing-platforms/hiseq-2500/specifications.html.

[70] Richards S., Aziz N., Bale S., Bick D., Das S., Gastier-Foster J., Grody W.W., Hegde M., Lyon E., Spector E. (2015). Standards and Guidelines for the Interpretation of Sequence Variants: A Joint Consensus Recommendation of the American College of Medical Genetics and Genomics and the Association for Molecular Pathology. Genet. Med..

[71] Fadista J., Oskolkov N., Hansson O., Groop L. (2017). LoFtool: A Gene Intolerance Score Based on Loss-of-Function Variants in 60,706 Individuals. Bioinformatics.

[72] Frésard L., Montgomery S.B. (2018). Diagnosing Rare Diseases after the Exome. Cold Spring Harb. Mol. Case Stud..

[73] Yao K., Dou B., Zhang Y., Chen Z., Li Y., Fan Z., Ma Y., Du S., Wang J., Xu Z. (2023). Inflammation-the Role of TRPA1 Channel. Front. Physiol..

[74] Señarís R., Ordás P., Reimúndez A., Viana F. (2018). Mammalian Cold TRP Channels: Impact on Thermoregulation and Energy Homeostasis. Pflugers Arch..

[75] Hellenthal K.E.M., Brabenec L., Gross E.R., Wagner N.M. (2021). TRP Channels as Sensors of Aldehyde and Oxidative Stress. Biomolecules.

[76] Irwin W.A., Bergamin N., Sabatelli P., Reggiani C., Megighian A., Merlini L., Braghetta P., Columbaro M., Volpin D., Bressan G.M. (2003). Mitochondrial Dysfunction and Apoptosis in Myopathic Mice with Collagen VI Deficiency. Nat. Genet..

[77] Okamoto Y., Takashima H. (2023). The Current State of Charcot-Marie-Tooth Disease Treatment. Genes.

[78] Barro-Soria R. (2023). Sensing Its Own Permeant Ion: KCNQ1 Channel Inhibition by External K. J. Gen. Physiol..

[79] Sheline C.T., Choi E.H., Kim-Han J.S., Dugan L.L., Choi D.W. (2002). Cofactors of Mitochondrial Enzymes Attenuate Copper-Induced Death In Vitro and In Vivo. Ann. Neurol..

[80] Delprat B., Rieusset J., Delettre C. (2019). Defective Endoplasmic Reticulum–Mitochondria Connection Is a Hallmark of Wolfram Syndrome. Contact.

[81] Lischka A., Lassuthova P., Çakar A., Record C.J., Van Lent J., Baets J., Dohrn M.F., Senderek J., Lampert A., Bennett D.L. (2022). Genetic Pain Loss Disorders. Nat. Rev. Dis. Prim..

[82] Hardiman O., Al-Chalabi A., Chio A., Corr E.M., Logroscino G., Robberecht W., Shaw P.J., Simmons Z., Van Den Berg L.H. (2017). Amyotrophic Lateral Sclerosis. Nat. Rev. Dis. Prim..

