# Supplementary material for: Broadening the Genetic Spectrum of Painful Small-Fiber Neuropathy through Whole-Exome Study in Early-Onset Cases
Source: Int J Mol Sci. 2024 Jun 30;25(13):7248. doi: 10.3390/ijms25137248 (PMC11242789; doi:10.3390/ijms25137248)
Supplement: Supplementary file 1 [file ijms-25-07248-s001.zip › Supplementary_Data.docx]

Supplementary

**Supplementary Data**

In-house analysis workflow

The raw reads, or fastq files, were used as input for the whole-exome sequencing (WES) analysis workflow. Initially, the quality of these raw reads was assessed with FastQC (version 0.11.8), evaluating aspects such as per-base sequence quality, sequence duplication levels, over-represented sequences, per sequence GC content, and per sequence quality scores. Next, adapters were removed from each read using Trimmomatic (version 0.39) to eliminate low-quality bases and adapters, followed by Cutadapt (version 2.7) to remove any remaining adapter sequences while maintaining read quality. The trimmed fastq files were then aligned to the human reference genome (GRCh37/hg19) using the Burrows-Wheeler Aligner-MEM (version 0.7.17). The aligned reads were converted to Binary Alignment Map (BAM) files with Samtools (version 1.9). These BAM files were further processed with Samtools and Picard (version 2.22.5) to generate alignment and coverage reports for each sample. Subsequently, the BAM files were processed using the Genome Analysis Toolkit (GATK) workflow (version 4.1.7) to identify variants. These variants were annotated using the SnpEff suite (version 4.3t), producing an annotated Variant Call Format (VCF) file. Several filtering steps were then applied to select single nucleotide variants (SNVs) and short insertions and deletions (INDELs) with potential deleterious effects. The VCF file was converted to a tabulated text file for filtering based on criteria such as high or moderate impact on protein translation and RNA splicing, sequencing depth quality of ≥10, alternate allele depth percentage of ≥ 25%, a GATK quality score of ≥100, alternate allele frequency of ≤ 5% or absence in the GnomAD population database for the non-Finnish European population, absence of the alternate allele in the Italian healthy controls, and a Phylop conservation score across 100 vertebrates of ≥ 2 or an undefined score.

Resultant variant summary statistics:

Total number of patients: 88

Total variants after application of variant filtering step: 54397

Total varints after application of gene filter step: 2424

Average variants per case: 27.5

Variant Types:

- SNPs: 1405

- Insertions: 450

- Deletions: 569

**Supplementary Figures**


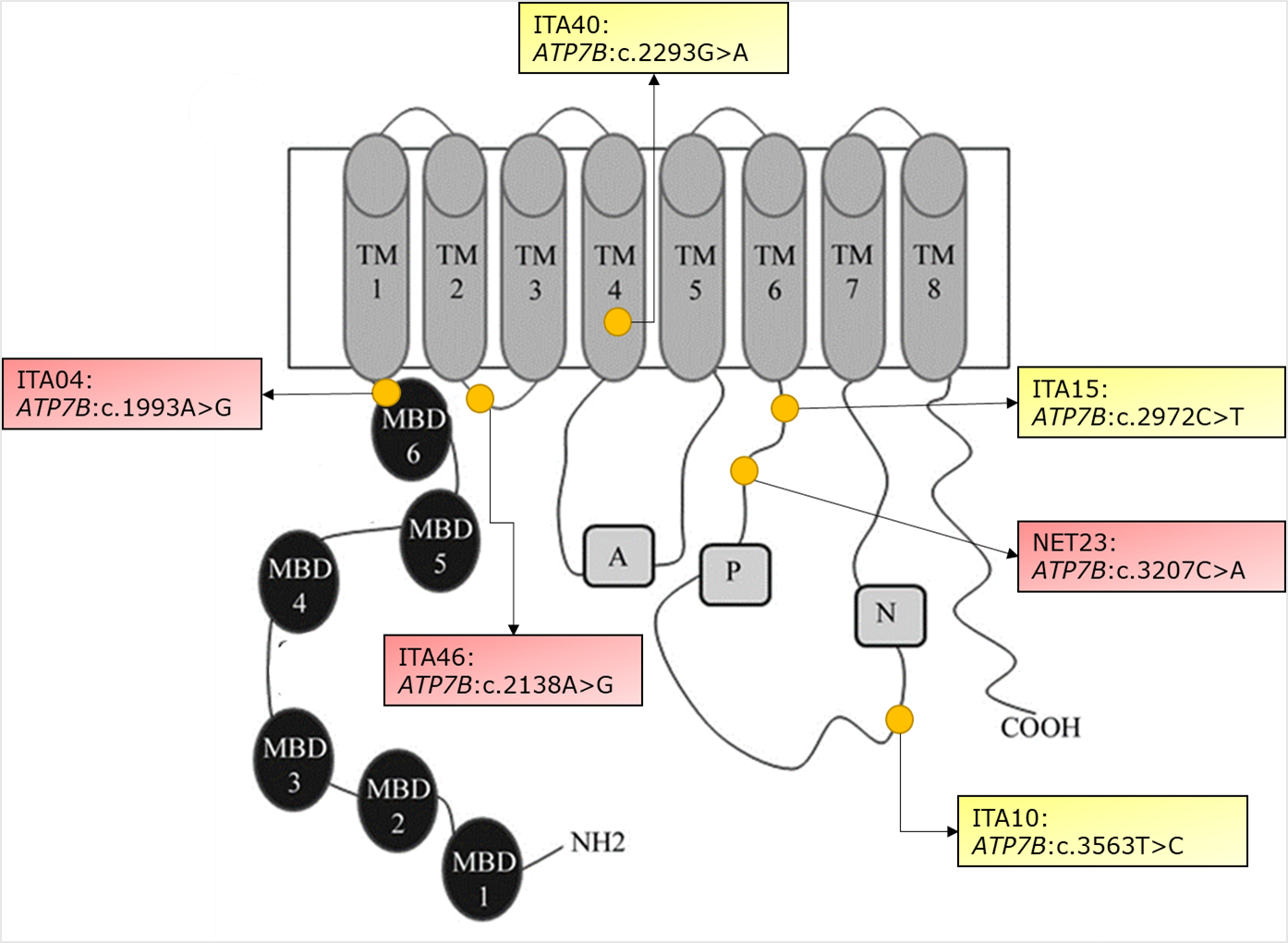


**Figure S1.** ATP7B protein structure consists of eight transmembrane segments (TM1-TM8) with NH2 denoting the amino side of the protein and COOH the carboxyl side. The circle with MBD represents metal-binding domains (MBD1-MBD6) at the N-terminus. The squares represent other domains: the A-actuator domain, the P-phosphorylation domain, and the N-nucleotide binding domain. The location of genetic variants observed in our cohort is displayed with yellow circles within the protein structure. The genetic variants classified as LP are shaded in red while the VUS variants are shaded in yellow along with their patient identifier ‘ITA’ for Italy and ‘NET’ for Netherlands.


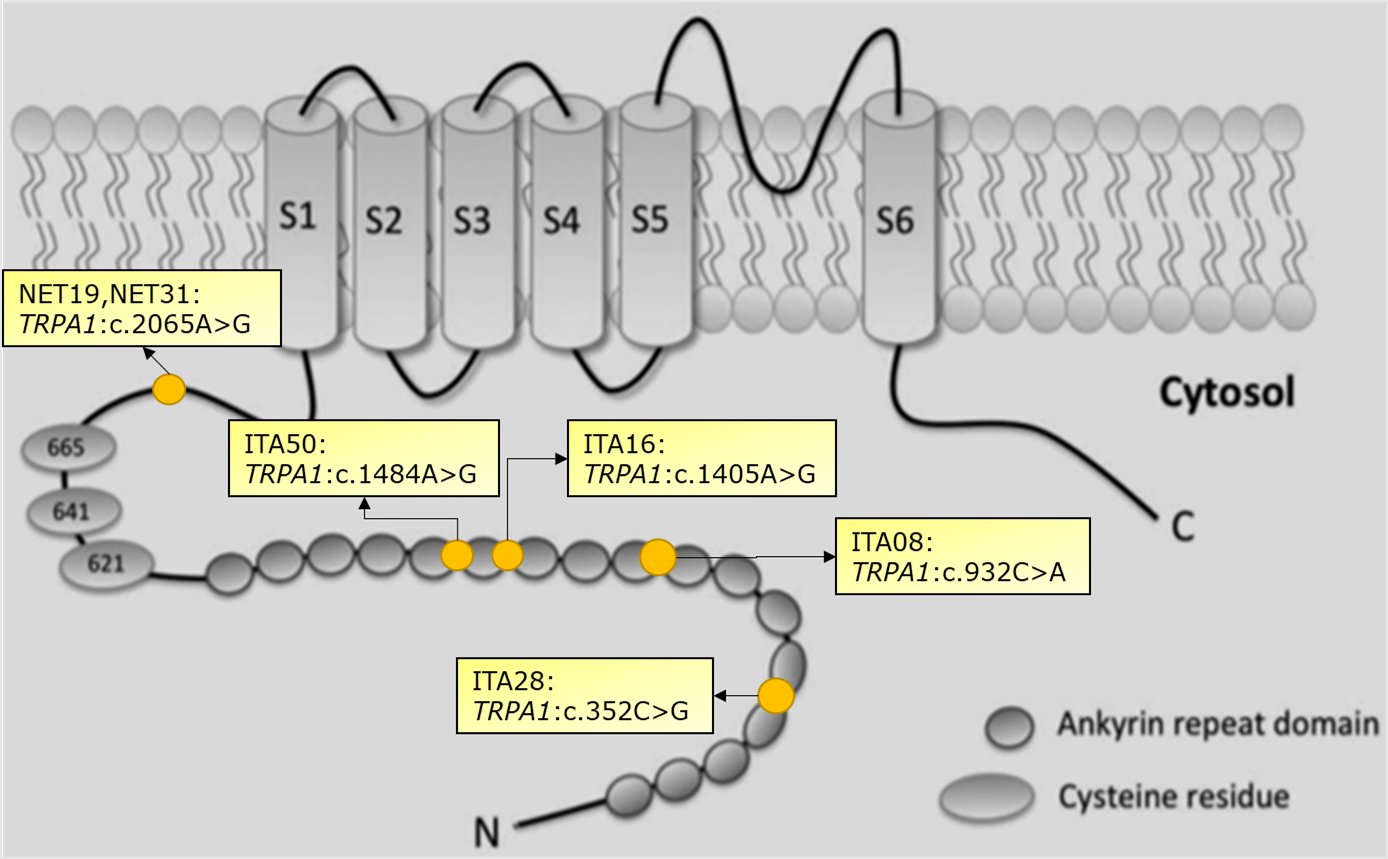


**Figure S2.** TRPA1 protein structure consisting of six transmembrane segments (S1-S6) with NH2 denoting the amino side of the protein and COOH the carboxyl side. The circle with a dark shade represents the ankyrin repeat domain at the N-terminus. Circle with numbers and lighter shade represents cysteine residues. The location of genetic variants observed in our cohort is displayed with yellow circles within the protein structure. The VUS variants are shaded in yellow along with their patient identifier ‘ITA’ for Italy and ‘NET’ for Netherlands.
